# Elotuzumab, pomalidomide, and dexamethasone is a very well tolerated regimen associated with durable remission even in very advanced myeloma: a retrospective study from two academic centers

**DOI:** 10.1007/s00432-020-03323-6

**Published:** 2020-07-18

**Authors:** Dorothea Hose, Martin Schreder, Jochen Hefner, Max Bittrich, Sophia Danhof, Susanne Strifler, Maria-Theresa Krauth, Renate Schoder, Bettina Gisslinger, Hermann Einsele, Heinz Gisslinger, Stefan Knop

**Affiliations:** 1 Division of Hematology, Wuerzburg University Medical Center, Würzburg, Germany; 2 Division of Psychosomatic Medicine, Wuerzburg University Medical Center, Würzburg, Germany; 3 Department of Hematology and Hemostaseology, Medical University Vienna, Vienna, Austria

**Keywords:** Multiple myeloma, Elotuzumab, SLAMF7, Pomalidomide, Lenalidomide

## Abstract

**Background:**

The anti-SLAMF7 monoclonal antibody, elotuzumab (elo), plus lenalidomide (len) and dexamethasone (dex) is approved for relapsed/refractory MM in the U.S. and Europe. Recently, a small phase 2 study demonstrated an advantage in progression-free survival (PFS) for elo plus pomalidomide (pom)/dex compared to pom/dex alone and resulted in licensing of this novel triplet combination, but clinical experience is still limited.

**Purpose:**

To analyze the efficacy and safety of elo/pom/dex in a “real world” cohort of patients with advanced MM, we queried the databases of the university hospitals of Würzburg and Vienna.

**Findings:**

We identified 22 patients with a median number of five prior lines of therapy who received elo/pom/dex prior to licensing within an early access program. Patients received a median number of 5 four-week treatment cycles. Median PFS was 6.4 months with 12-month and 18-month PFS rates of 35% and 28%, respectively. The overall response rate was 50% and 64% of responding patients who achieved a longer PFS with elo/pom/dex compared to their most recent line of therapy. Objective responses were also seen in five patients who had been pretreated with pomalidomide. Low tumor burden was associated with improved PFS (13.5 months for patients with ISS stage I/II at study entry v 6.4 months for ISS III), although this difference did not reach statistical significance. No infusion-related reactions were reported. The most frequent grade 3/4 adverse events were neutropenia and pneumonia.

**Conclusion:**

Elo/pom/dex is an active and well-tolerated regimen in highly advanced MM even after pretreatment with pomalidomide.

## Introduction

Multiple Myeloma (MM) is the second most frequent hematologic malignancy in the U.S. and Europe (Rollig et al. 2015). It is characterized by an uncontrolled proliferation of clonal plasma cells in the bone marrow and the accumulation of abnormal intact or incomplete immunoglobulins in serum and/or urine (Moreau et al. 2017; Raab et al. 2009). The median age at diagnosis of MM is 69 years with most subjects being diagnosed above the age of 55 years and a male predominance (Raab et al. 2016). Advances in therapeutic strategies have led to an increase in median overall survival of patients from three to six years within the last two decades, owing to novel compounds like proteasome inhibitors (PIs, e.g. bortezomib, carfilzomib, ixazomib) immunomodulatory drugs (IMIDs, e.g. thalidomide, lenalidomide, pomalidomide), alkylating agents (e.g. melphalan) or histone deacetylase inhibitors (e.g. panobinostat). Multi-drug combinations improve the long-term treatment outcome and might overcome drug resistance (Schreder and Knop 2019), but most patients continue to have relapses and will eventually become refractory to available drugs. Every subsequent relapse induces a shortened progression-free interval (Yong et al. 2016); therefore, novel treatment approaches are needed.

Immunotherapy holds great promise for MM therapy. MoAbs selectively target antigens on the myeloma cell surface which are critical for signaling, tumor growth, and survival (van de Donk et al. 2016). Elotuzumab is a humanized monoclonal IgGκ-antibody targeting the signaling lymphocytic activation molecule F7 (SLAMF7) or CS1 (CD2 subset-1), a glycoprotein universally and highly expressed on the surface of normal and malignant plasma cells as well as natural killer cells (Einsele and Schreder 2016). Elotuzumab) exhibited significant in vitro antibody-dependent cellular cytotoxicity (ADCC) using primary myeloma cells as targets and both allogeneic and autologous NK cells as effectors. Furthermore, in vivo assays showed antitumor activity, which depended on efficient Fc-CD16 interaction as well as the presence of NK cells in mice (Hsi et al. 2008). The specificity enables elotuzumab to selectively kill myeloma cells and induce minimal damage on healthy tissue. In a randomized phase III trial, the addition of elotuzumab to lenalidomide and low-dose dexamethasone (Rd) resulted in a sustained improvement of progression-free survival (PFS) compared to Rd, leading to approval of the triplet regimen by the FDA and EMA (Lonial et al. 2015). However, patients who were refractory or intolerant to lenalidomide were excluded from the registration trial.

As pomalidomide is known to induce objective responses in len-refractory patients, we substituted lenalidomide for pomalidomide in patients with very advanced MM who were otherwise eligible for treatment with elotuzumab, dexamethasone and an IMID. Meanwhile, the results of a small randomized phase II study comparing elotuzumab/pomalidomide/dexamethasone (elo/pom/dex) with pom/dex have been reported, demonstrating a high efficacy of the triplet regimen in patients with relapsed/refractory MM (Dimopoulos et al. 2018). Here, we present the outcome of 22 consecutive MM patients with very advanced disease who received the triplet combination of elo/pom/dex outside of a clinical trial at two tertiary care centers.

## Methods

We queried the databases of the university hospitals of Würzburg and Vienna to identify patients with relapsed and refractory multiple myeloma receiving elo/pom/dex in an individualized treatment concept when no other option was available. Patients had to have measurable disease according to the IMWG criteria (Rajkumar et al. 2014). Pretreatment with pomalidomide was allowed, but patients refractory to the compound were excluded. All patients provided written informed consent.

Elotuzumab was given intravenously at a dose of 10 mg/kg bodyweight on days 1, 8, 15 and 22 of a 28-day cycle in cycles 1 and 2 and on days 1 and 15 in subsequent cycles. Pomalidomide was administered orally at a dose of 4 mg on days 1 through 21 of each cycle. Dexamethasone was given weekly at a dose of 28 mg orally plus 8 mg intravenously on elotuzumab treatment days and 40 mg orally in weeks without elotuzumab. Dose reductions of pomalidomide and dexamethasone were performed in the event of toxicities according to the SmPC. Patients received antimicrobial prophylaxis with aciclovir and cotrimoxazole as well as low molecular weight heparin or acetylsalicylic acid (aspirin^®^) as prophylaxis of thromboembolic events throughout the treatment period. Treatment was continued until disease progression (PD) or unacceptable toxicity. Responses were defined according to IMWG criteria (Kumar et al. 2016). Adverse events (AEs) were recorded and graded according to the National Cancer Institute Common Terminology Criteria for Adverse Events, v. 4.0.

The database was locked on 1st February 2020. Statistical analysis was done with IBM SPSS statistics (IBM, Ehningen, Germany) and Prism (Graph Pad Software, San Diego, CA, USA). PFS was calculated according to Kaplan–Meier method from first dose of elo/pom/dex to disease progression or death, whatever occurred first. Patients proceeding to an autologous stem cell transplantation (SCT) were censored at time of transplant. Overall survival (OS) was calculated from first dose of elo/pom/dex to death from any cause or loss of follow up.

## Results

### Patients

We identified 22 patients with a median age of 61.5 years (range 39–81); the median duration of myeloma was 6.7 years (range 0.3–11.7). The baseline characteristics of the study population are shown in Table 1. The first patients began treatment in October 2015 and the last patient was started on elo/pom/dex in January 2017. A baseline bone marrow (BM) biopsy was performed in 18 patients. The median plasma cell infiltration was 35% (range 5–90) and 4 patients (18%) had high-risk cytogenetic abnormalities. The median number of prior treatment lines was 5 (range 1–16). All patients had been exposed to bortezomib and lenalidomide, 18 (82%) had previously undergone a stem cell transplantation. Only two patients had received prior daratumumab and five patients had been exposed to carfilzomib. Fifteen patients (68%) had been treated with pomalidomide during any previous regimen; of these, ten patients had received pomalidomide in the most recent line of therapy and were consecutively switched to elo/pom/dex after failing to achieve an objective response. 13 patients (59%) had been refractory to both their most recent line of therapy and earlier lenalidomide.

**Table 1:** Clinical characteristics of patients at baseline

Characteristic	Value (*n* = 22)
Age, median (range), years	61.5 (39–81)
Male sex, *n *(%)	16 (73)
Type of myeloma, *n *(%)	
IgG	13 (59)
IgA	5 (23)
IgD	1 (4)
Light chain	3 (14)
International Staging System (ISS) stage at study entry, *n *(%)	
I–II	13 (59)
III	5 (23)
Missing data	4 (18)
BM plasma cell infiltration, (%)	
< 30%	7 (32)
30–59%	6 (27)
≥ 60	5 (23)
Not reported	4 (18)
Cytogenetic abnormality, *n* (%)	
del17p, t(4;14), or t(14;16)	
Yes	4 (18)
No	15 (68)
Data not available	
1q21	3 (14)
Yes	2 (9)
No	16 (73)
Data not available	4 (18)
Primary refractory to first line treatment, *n *(%)	7 (32)
Median No. of previous treatment regimens (range)	5 (1–16)
Median time since initial diagnosis (range), years	6.7 (0.3–11.7)
Prior autologous stem cell transplantation, *n *(%)	17 (77)
Prior allogeneic stem cell transplantation, *n *(%)	1 (4)
Prior pomalidomide, *n *(%)	15 (68)
Prior carfilzomib, *n *(%)	5 (23)
Prior daratumumab, *n *(%)	2 (9)
Refractory to most recent line of therapy, *n *(%)	13 (59)
Refractory to lenalidomide, *n *(%)	13 (59)

### Treatment exposure

At database lock all patients had discontinued treatment, with disease progression as the most common reason. Only one patient discontinued early due to side effects. Two patients requested to stop IV treatment with elotuzumab after 6 and 10 cycles of the triplet combination, respectively, and continued on pom/dex. Another two patients went on to receive an autologous stem cell transplantation as a means of consolidation. The median number of treatment cycles was 5 (range 1–30).

### Efficacy

All 22 patients were evaluable for response. 11 patients achieved a partial response (PR), yielding an overall response rate of 50%. Of note, five of these patients had been primary refractory to their first line regimen.

**Fig. 1:**
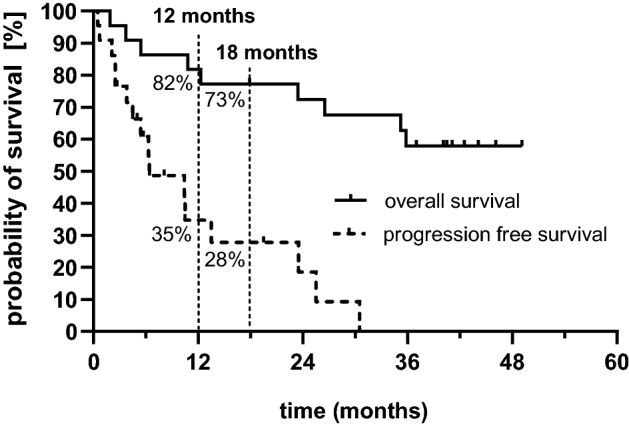
Progression-free (dashed line) and overall survival (solid line) with elotuzumab, pomalidomide, and dexamethasone. PFS and OS rates at 12 and 18 months from start of treatment are displayed

**Fig. 2:**
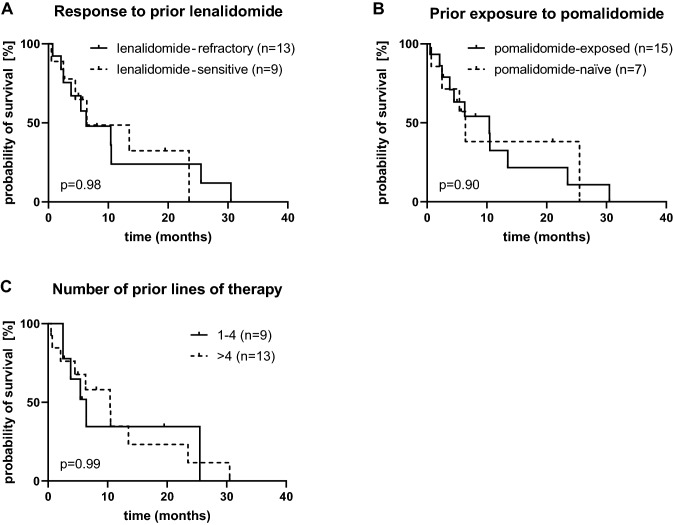
Progression-free survival with elotuzumab/pomalidomide/dexamethasone according to **a** response to prior lenalidomide, **b** prior exposure tp pomalidomide and **c** number of prior lines of therapy

The median PFS was 6.4 months. In a landmark analysis, 35% and 28% of patients were progression-free at 12 months and 18 months, respectively (Figs. 1, 2). Patients with high-risk cytogenetics had identical PFS compared to those with standard-risk disease (6.5 v 6.4 months, *p* = 0.77). There was a clear trend for shorter PFS in patients with ISS stage III at study entry compared to those with stage I and II disease (6.5 vs. 13.5 months), which did not reach statistical significance due to small sample size.

Patients refractory to lenalidomide showed no difference in their PFS compared to non-refractory patients (*p* = 0.98, Fig. 2a). Among patients who had previously received pomalidomide, 5 (33%) responded and another 3 (20%) had stable disease with most responses seen in patients who had pomalidomide immediately prior to elo/pom/dex (4 PR, 2 SD). Median PFS in pomalidomide-exposed patients was identical to that seen in pom-naïve patients (*p* = 0.90, Fig. 2b). When elo/pom/dex was given directly after a pomalidomide-containing regimen (e.g., carfilzomib/pom/dex, bortezomib/doxorubicin/pom/dex), an absolute gain in PFS of 4.3 months (*p* = 0.192) was seen when compared to patients with a regimen that did not include pomalidomide in the preceding line.

Responses were also observed in 3 out of 5 patients who had been pretreated with a carfilzomib-based regimen; PFS did not differ significantly when compared to carfilzomib-naïve subjects. At database lock, 14 of 22 patients (64%) had achieved a longer PFS to elo/pom/dex when compared to their most recent line of therapy.

PFS did not differ in intensely pretreated (> 4 prior therapies) versus less heavily pretreated patients (*p* = 0.99, Fig. 2c).

The median follow-up for the study population was 42.5 months. The median overall survival (95% CI) was not reached (23.6 months—not estimable). At 12 months, 82% of patients (*n* = 18) were still alive; the 18-month OS rate was 73% (Fig. 1).

In total, 17 of 21 patients (81%) received subsequent systemic therapy. One patient was lost to follow-up after discontinuation of elo/pom/dex. Two thirds of patients were treated with a daratumumab-containing regimen (*n* = 14); median exposure to the anti-CD38 antibody was 13.5 months. 4 patients underwent a salvage autologous stem cell transplant, two immediately after elo/pom/dex and another two later in the course of their disease. Other alkylating agents (most commonly, bendamustine or cyclophosphamide) were used in 57% and carfilzomib-based combinations in 43% of patients, respectively.

### Toxicity

No infusion-related reactions were observed. In three patients, grade 3/4 neutropenia was recorded. In two of them the absolute neutrophil count dropped below 500/µl (grade 4), but no patient experienced neutropenic fever. One patient had grade 3 thrombocytopenia following the accidental continuous intake of pomalidomide.

Four patients were diagnosed with a grade 3/4 respiratory infection, two of whom sustained a pneumonia grade 3. Streptococcus pneumoniae was isolated from one patient and parainfluenza II virus in another patient; in the remaining cases, the offending pathogen could not be identified. All patients resumed treatment after resolution of symptoms.

## Discussion

In recent years, immunotherapy has attracted significant attention in the treatment of relapsed or refractory MM. One milestone was the pivotal phase 2 study of daratumumab demonstrating single agent activity in patients with PI- and IMiD-refractory MM with a 3.7 months PFS and a median OS of 17.5 months. Meanwhile, daratumumab-based regimens are widely used in relapsed disease (Dimopoulos et al. 2016a; Mateos et al. 2020) and have recently been approved for frontline therapy in both transplant-eligible (Moreau et al. 2019a) and transplant-ineligible patients (Facon et al. 2019; Mateos et al. 2018).

While many patients will now receive CD38 antibody-based treatment, immunotherapy directed at alternative antigens are needed. Elotuzumab targets SLAMF7, acts synergistically with IMiDs and was shown to induce durable remissions in relapsed MM with a PFS of 19.4 months when combined with len/dex (Lonial et al. 2015). However, most patients now receive len as part of their first-line regimen and a considerable fraction will develop len-resistant disease (Moreau et al. 2019b). In this setting, pomalidomide/dex is active with a modest PFS of 4.0 months in the registration trial (Dimopoulos et al. 2016b), but long-lasting remissions are also observed (Danhof et al. 2015). The addition of elotuzumab to pom/dex aiming at prolonged disease control appears tempting and was proven effective in a randomized phase II trial (Dimopoulos et al. 2018). This study reported a median PFS of 10.3 months with elo/pom/dex in patients with a median number of 3 prior lines of therapy.

Since only 60 patients were included in the experimental arm of the ELOQUENT-3 study, we sought to expand clinical experience with elo/pom/dex in a “real-world” cohort of advanced MM. Compared to the published dataset, the 22 subjects of our current retrospective analysis had a longer interval since diagnosis of their MM (6.7 vs 4.8 years) and were more heavily pretreated (median, 5 vs 3 prior lines). Remarkably, 32% (*n* = 7) of them had been primary refractory to first-line treatment; 59% (*n* = 13) were refractory to lenalidomide and two thirds had previously received pomalidomide.

In this unfavorable cohort, we were still able to demonstrate a median PFS of 6.4 months with PFS rates at 12 and 18 months that were comparable to those reported in a recent update of ELOQUENT-3 (35% and 28% vs 43% and 34%, respectively) (Dimopoulos 2019). An overall response rate of 50% compared equally well to the published data. Of note, 64% of subjects achieved a longer PFS when compared to their most recent line of therapy and we were able to confirm responses in pomalidomide-exposed patients, most of whom had received pomalidomide in the most recent line prior to elo/pom/dex. This observation would thus justify the addition of elotuzumab to a doublet regimen of pom/dex in the absence of frank progression which in our hands led to objective responses in 4 of 10 patients. Incremental gain of median PFS with Elo/Pd was 4.3 months when compared to PFS with the most recent line of therapy. We believe this constitutes a clinically relevant benefit, as median PFS in a very advanced patient cohort was recently reported to be 3.4 months (Gandhi et al. 2019).

Even though none of our patients reached a complete remission (CR), we observed remarkably durable remissions of more than 20 months in 4 patients. In another two patients, elo/pom/dex served as a bridging therapy to autologous stem cell transplantation.

Subgroup analyses were limited due to the small sample size. Neither cytogenetic risk nor the number and type of prior therapy predicted for outcome in our cohort. Patients with low tumor burden as defined by ISS stages I and II at start of treatment appeared to gain increased benefit compared to those with stage III disease, confirming our previous observation (Danhof et al. 2019). We could also demonstrate that highly pretreated patients (> 4 prior therapies) showed a similar PFS compared to patients with a lower number of previous therapies, justifying the use of this regimen even in late stage MM.

In terms of toxicity, no new safety signals were seen. In general, treatment with elo/pom/dex was well tolerated; there was no allergic reaction or other infusion-related reaction recorded.

In one case a drug-related rash grade 2 was reported, diminishing under ongoing treatment. Like in many reported trials in advanced MM, respiratory infections were among the most common adverse events and were found to be severe in four patients, two of them presenting with grade 3 pneumonia. Both patients could resume therapy and achieved a PR. Hematologic toxicity was low with only a small number of patients experiencing grade 3/4 neutropenia. Grade 3 thrombocytopenia in one patient could be attributed to accidental continuous intake of pomalidomide. Taken together, adverse event rates were comparable to those reported in the ELOQUENT-3 trial.

Not least due to the favurable safety profile, 81% of our patients were able to receive subsequent systemic treatment upon progression on elo/pom/dex. Like in ELOQUENT-3, all but 2 patients had not been exposed to an anti-CD38 antibody and received daratumumab-based regimens for a median duration of more than one year. Overall survival rates of 86% and 81% at 12 and 18 months, respectively, are profoundly remarkable for heavily pretreated MM patients and are compare positively with published results. However, many patients with late-stage disease will now be pretreated with daratumumab; as in our study, these are largely underrepresented in current trials e.g. with pomalidomide-based combinations (Attal et al. 2019; Richardson et al. 2019) and will represent a formidable therapeutic challenge in the near future. A recent trial reported responses in 48% of daratumumab-exposed patients and acceptable toxicity with a quadruplet regimen of elo/pom/dex and bortezomib (Yee 2019), further corroborating our findings.

In summary, despite the small number of patients included here, our results suggest the combination of elo/pom/dex to represent an effective and exceptionally well-tolerated option in the treatment of advanced MM that may be considered in the len-refractory or even pom-exposed patient.

## Data Availability

The data generated and used for this study, are included in the published article. Additional data are available from the authors upon reasonable and individual request.

## References

[CR1] Attal M, Richardson PG, Rajkumar SV, San-Miguel J, Beksac M, Spicka I, Leleu X, Schjesvold F, Moreau P, Dimopoulos MA, Huang JS, Minarik J, Cavo M, Prince HM, Mace S, Corzo KP, Campana F, Le-Guennec S, Dubin F, Anderson KC, Icaria-Mm study group (2019) Isatuximab plus pomalidomide and low-dose dexamethasone versus pomalidomide and low-dose dexamethasone in patients with relapsed and refractory multiple myeloma (ICARIA-MM): a randomised, multicentre, open-label, phase 3 study. Lancet 394:2096–2107 31735560 10.1016/S0140-6736(19)32556-5

[CR2] Danhof S, Schreder M, Strifler S, Einsele H, Knop S (2015) Long-term disease control by pomalidomide-/dexamethasone-based therapy in a patient with advanced multiple myeloma: a case report and review of the literature. Case Rep Oncol 8:189–195 25969681 10.1159/000381983 PMC4427147

[CR3] Danhof S, Strifler S, Hose D, Kortum M, Bittrich M, Hefner J, Einsele H, Knop S, Schreder M (2019) Clinical and biological characteristics of myeloma patients influence response to elotuzumab combination therapy. J Cancer Res Clin Oncol 145:561–571 30519736 10.1007/s00432-018-2807-1 PMC11810234

[CR4] Dimopoulos M (2019) Elotuzumab plus pomalidomide and dexamethasone for relapsed/refractory multiple myeloma: efficacy results after additional follow-up of the phase 2, randomized ELOQUENT-3 study. Hemasphere 3:626–627

[CR5] Dimopoulos MA, Oriol A, Nahi H, San-Miguel J, Bahlis NJ, Usmani SZ, Rabin N, Orlowski RZ, Komarnicki M, Suzuki K, Plesner T, Yoon SS, Ben Yehuda D, Richardson PG, Goldschmidt H, Reece D, Lisby S, Khokhar NZ, O'Rourke L, Chiu C, Qin X, Guckert M, Ahmadi T, Moreau P, Investigators P (2016a) Daratumumab, lenalidomide, and dexamethasone for multiple myeloma. N Engl J Med 375:1319–1331 27705267 10.1056/NEJMoa1607751

[CR6] Dimopoulos MA, Palumbo A, Corradini P, Cavo M, Delforge M, Di Raimondo F, Weisel KC, Oriol A, Hansson M, Vacca A, Blanchard MJ, Goldschmidt H, Doyen C, Kaiser M, Petrini M, Anttila P, Cafro AM, Raymakers R, San-Miguel J, de Arriba F, Knop S, Rollig C, Ocio EM, Morgan G, Miller N, Simcock M, Peluso T, Herring J, Sternas L, Zaki MH, Moreau P (2016b) Safety and efficacy of pomalidomide plus low-dose dexamethasone in STRATUS (MM-010): a phase 3b study in refractory multiple myeloma. Blood 128:497–503 27226434 10.1182/blood-2016-02-700872 PMC5009959

[CR7] Dimopoulos MA, Dytfeld D, Grosicki S, Moreau P, Takezako N, Hori M, Leleu X, LeBlanc R, Suzuki K, Raab MS, Richardson PG, Popa McKiver M, Jou YM, Shelat SG, Robbins M, Rafferty B, San-Miguel J (2018) Elotuzumab plus pomalidomide and dexamethasone for multiple myeloma. N Engl J Med 379:1811–1822 30403938 10.1056/NEJMoa1805762

[CR8] Einsele H, Schreder M (2016) Treatment of multiple myeloma with the immunostimulatory SLAMF7 antibody elotuzumab. Ther Adv Hematol 7:288–301 27695618 10.1177/2040620716657993 PMC5026292

[CR9] Facon T, Kumar S, Plesner T, Orlowski RZ, Moreau P, Bahlis N, Basu S, Nahi H, Hulin C, Quach H, Goldschmidt H, O'Dwyer M, Perrot A, Venner CP, Weisel K, Mace JR, Raje N, Attal M, Tiab M, Macro M, Frenzel L, Leleu X, Ahmadi T, Chiu C, Wang J, Van Rampelbergh R, Uhlar CM, Kobos R, Qi M, Usmani SZ, Investigators MT (2019) Daratumumab plus lenalidomide and dexamethasone for untreated myeloma. N Engl J Med 380:2104–2115 31141632 10.1056/NEJMoa1817249 PMC10045721

[CR10] Gandhi UH, Cornell RF, Lakshman A, Gahvari ZJ, McGehee E, Jagosky MH, Gupta R, Varnado W, Fiala MA, Chhabra S, Malek E, Mansour J, Paul B, Barnstead A, Kodali S, Neppalli A, Liedtke M, Narayana S, Godby KN, Kang Y, Kansagra A, Umyarova E, Scott EC, Hari P, Vij R, Usmani SZ, Callander NS, Kumar SK, Costa LJ (2019) Outcomes of patients with multiple myeloma refractory to CD38-targeted monoclonal antibody therapy. Leukemia 33:2266–2275 30858549 10.1038/s41375-019-0435-7 PMC6820050

[CR11] Hsi ED, Steinle R, Balasa B, Szmania S, Draksharapu A, Shum BP, Huseni M, Powers D, Nanisetti A, Zhang Y, Rice AG, van Abbema A, Wong M, Liu G, Zhan F, Dillon M, Chen S, Rhodes S, Fuh F, Tsurushita N, Kumar S, Vexler V, Shaughnessy JD Jr, Barlogie B, van Rhee F, Hussein M, Afar DE, Williams MB (2008) CS1, a potential new therapeutic antibody target for the treatment of multiple myeloma. Clin Cancer Res 14:2775–2784 18451245 10.1158/1078-0432.CCR-07-4246 PMC4433038

[CR12] Kumar S, Paiva B, Anderson KC, Durie B, Landgren O, Moreau P, Munshi N, Lonial S, Bladé J, Mateos M-V, Dimopoulos M, Kastritis E, Boccadoro M, Orlowski R, Goldschmidt H, Spencer A, Hou J, Chng WJ, Usmani SZ, Zamagni E, Shimizu K, Jagannath S, Johnsen HE, Terpos E, Reiman A, Kyle RA, Sonneveld P, Richardson PG, McCarthy P, Ludwig H, Chen W, Cavo M, Harousseau J-L, Lentzsch S, Hillengass J, Palumbo A, Alberto Orfao S, Rajkumar V, Miguel JS, Avet-Loiseau H (2016) International Myeloma Working Group consensus criteria for response and minimal residual disease assessment in multiple myeloma. Lancet Oncol 17:e328–e346 27511158 10.1016/S1470-2045(16)30206-6

[CR13] Lonial S, Dimopoulos M, Palumbo A, White D, Grosicki S, Spicka I, Walter-Croneck A, Moreau P, Mateos MV, Magen H, Belch A, Reece D, Beksac M, Spencer A, Oakervee H, Orlowski RZ, Taniwaki M, Rollig C, Einsele H, Wu KL, Singhal A, San-Miguel J, Matsumoto M, Katz J, Bleickardt E, Poulart V, Anderson KC, Richardson P, Investigators E (2015) Elotuzumab therapy for relapsed or refractory multiple myeloma. N Engl J Med 373:621–631 26035255 10.1056/NEJMoa1505654

[CR14] Mateos MV, Dimopoulos MA, Cavo M, Suzuki K, Jakubowiak A, Knop S, Doyen C, Lucio P, Nagy Z, Kaplan P, Pour L, Cook M, Grosicki S, Crepaldi A, Liberati AM, Campbell P, Shelekhova T, Yoon SS, Iosava G, Fujisaki T, Garg M, Chiu C, Wang J, Carson R, Crist W, Deraedt W, Nguyen H, Qi M, San-Miguel J, Investigators AT (2018) Daratumumab plus bortezomib, melphalan, and prednisone for untreated myeloma. N Engl J Med 378:518–528 29231133 10.1056/NEJMoa1714678

[CR15] Mateos MV, Cavo M, Blade J, Dimopoulos MA, Suzuki K, Jakubowiak A, Knop S, Doyen C, Lucio P, Nagy Z, Pour L, Cook M, Grosicki S, Crepaldi A, Liberati AM, Campbell P, Shelekhova T, Yoon SS, Iosava G, Fujisaki T, Garg M, Krevvata M, Chen Y, Wang J, Kudva A, Ukropec J, Wroblewski S, Qi M, Kobos R, San-Miguel J (2020) Overall survival with daratumumab, bortezomib, melphalan, and prednisone in newly diagnosed multiple myeloma (ALCYONE): a randomised, open-label, phase 3 trial. Lancet 395:132–141 31836199 10.1016/S0140-6736(19)32956-3

[CR16] Moreau P, San Miguel J, Sonneveld P, Mateos MV, Zamagni E, Avet-Loiseau H, Hajek R, Dimopoulos MA, Ludwig H, Einsele H, Zweegman S, Facon T, Cavo M, Terpos E, Goldschmidt H, Attal M, Buske C, Esmo Guidelines Committee (2017) Multiple myeloma: ESMO Clinical Practice Guidelines for diagnosis, treatment and follow-up. Ann Oncol 28:iv52–iv61 28453614 10.1093/annonc/mdx096

[CR17] Moreau P, Attal M, Hulin C, Arnulf B, Belhadj K, Benboubker L, Bene MC, Broijl A, Caillon H, Caillot D, Corre J, Delforge M, Dejoie T, Doyen C, Facon T, Sonntag C, Fontan J, Garderet L, Jie KS, Karlin L, Kuhnowski F, Lambert J, Leleu X, Lenain P, Macro M, Mathiot C, Orsini-Piocelle F, Perrot A, Stoppa AM, van de Donk NW, Wuilleme S, Zweegman S, Kolb B, Touzeau C, Roussel M, Tiab M, Marolleau JP, Meuleman N, Vekemans MC, Westerman M, Klein SK, Levin MD, Fermand JP, Escoffre-Barbe M, Eveillard JR, Garidi R, Ahmadi T, Zhuang S, Chiu C, Pei L, de Boer C, Smith E, Deraedt W, Kampfenkel T, Schecter J, Vermeulen J, Avet-Loiseau H, Sonneveld P (2019a) Bortezomib, thalidomide, and dexamethasone with or without daratumumab before and after autologous stem-cell transplantation for newly diagnosed multiple myeloma (CASSIOPEIA): a randomised, open-label, phase 3 study. Lancet 394:29–38 31171419 10.1016/S0140-6736(19)31240-1

[CR18] Moreau P, Zamagni E, Mateos MV (2019b) Treatment of patients with multiple myeloma progressing on frontline-therapy with lenalidomide. Blood Cancer J 9:38 30894516 10.1038/s41408-019-0200-1 PMC6426995

[CR19] Raab MS, Podar K, Breitkreutz I, Richardson PG, Anderson KC (2009) Multiple myeloma. Lancet 374:324–339 19541364 10.1016/S0140-6736(09)60221-X

[CR20] Raab MS, Lehners N, Xu J, Ho AD, Schirmacher P, Goldschmidt H, Andrulis M (2016) Spatially divergent clonal evolution in multiple myeloma: overcoming resistance to BRAF inhibition. Blood 127:2155–2157 26884375 10.1182/blood-2015-12-686782

[CR21] Rajkumar SV, Dimopoulos MA, Palumbo A, Blade J, Merlini G, Mateos M-V, Kumar S, Hillengass J, Kastritis E, Richardson P, Landgren O, Paiva B, Dispenzieri A, Weiss B, LeLeu X, Zweegman S, Lonial S, Rosinol L, Zamagni E, Jagannath S, Sezer O, Kristinsson SY, Caers Jo, Usmani SZ, Lahuerta JJ, Johnsen HE, Beksac M, Cavo M, Goldschmidt H, Terpos E, Kyle RA, Anderson KC, Durie BGM, San JF, Miguel (2014) International Myeloma Working Group updated criteria for the diagnosis of multiple myeloma. Lancet Oncol 15:e538–e548 25439696 10.1016/S1470-2045(14)70442-5

[CR22] Richardson PG, Oriol A, Beksac M, Liberati AM, Galli M, Schjesvold F, Lindsay J, Weisel K, White D, Facon T, San Miguel J, Sunami K, O'Gorman P, Sonneveld P, Robak P, Semochkin S, Schey S, Yu X, Doerr T, Bensmaine A, Biyukov T, Peluso T, Zaki M, Anderson K, Dimopoulos M, Optimismm trial investigators (2019) Pomalidomide, bortezomib, and dexamethasone for patients with relapsed or refractory multiple myeloma previously treated with lenalidomide (OPTIMISMM): a randomised, open-label, phase 3 trial. Lancet Oncol 20:781–794 31097405 10.1016/S1470-2045(19)30152-4

[CR23] Rollig C, Knop S, Bornhauser M (2015) Multiple myeloma. Lancet 385:2197–2208 25540889 10.1016/S0140-6736(14)60493-1

[CR24] Schreder M, Knop S (2019) Are triplet therapies really living up to their hype as the "standard of care" for multiple myeloma and what else is needed? Expert Rev Hematol 12:699–701 31287740 10.1080/17474086.2019.1641403

[CR25] van de Donk NW, Moreau P, Plesner T, Palumbo A, Gay F, Laubach JP, Malavasi F, Avet-Loiseau H, Mateos MV, Sonneveld P, Lokhorst HM, Richardson PG (2016) Clinical efficacy and management of monoclonal antibodies targeting CD38 and SLAMF7 in multiple myeloma. Blood 127:681–695 26631114 10.1182/blood-2015-10-646810

[CR26] Yee AL (2019) A phase II study of elotuzumab in combination with pomalidomide, bortezomib, and dexamethasone in relapsed and refractory multiple myeloma. Blood 134:316910.1182/bloodadvances.202401471739626297

[CR27] Yong K, Delforge M, Driessen C, Fink L, Flinois A, Gonzalez-McQuire S, Safaei R, Karlin L, Mateos MV, Raab MS, Schoen P, Cavo M (2016) Multiple myeloma: patient outcomes in real-world practice. Br J Haematol 175:252–264 27411022 10.1111/bjh.14213 PMC5096152

